# Prevalence of major external structural birth defects in Kiambu County, Kenya, 2014-2018

**DOI:** 10.11604/pamj.2020.37.187.26289

**Published:** 2020-10-28

**Authors:** George Nyadimo Agot, Marshal Mutinda Mweu, Joseph Kibuchi Wang’ombe

**Affiliations:** 1 School of Public Health, College of Health Sciences, University of Nairobi, Nairobi, Kenya

**Keywords:** Major, external, structural, birth, defects, prevalence, county

## Abstract

**Introduction:**

major external structural birth defects are typical and have been associated with childhood morbidity, mortality and lifelong resource-intensive disabilities. These defects continue to occur; however, they are yet to be recognized as public health problems in Kenya. The objective of this study was to estimate the prevalence of major external structural birth defects in Kiambu County in Kenya, 2014-2018.

**Methods:**

a cross-sectional study design was adopted; a retrospective review of medical records was conducted between 2014 and 2018 abstracting 873 birth defects. Following a predetermined inclusion criterion, a five-year prevalence numerator of 362 cases was determined, whereas, a five-year prevalence denominator of 299,854 cases of registered live-births was obtained from the birth registrar. Annual prevalence estimates of 29 sub-groups and 6 groups of these defects were calculated as the number of cases (numerator) divided by the number of live-births (denominator). Associated 95% binomial exact confidence intervals were also computed and expressed per 100,000 live-births.

**Results:**

defects of the musculoskeletal system, the central nervous system, orofacial, genital organs, eye and anus were observed. Defects of the musculoskeletal system were the most prevalent, ranging from 22.98 (95% CI: 11.87-40.13) to 116.9 (95% CI: 92.98-145.08) per 100,000 live-births. Defects of the central nervous system followed ranging between 13.40 (95% CI: 5.39-27.61) and 32.79 (95% CI: 20.79-49.19) per 100,000 live-births.

**Conclusion:**

despite musculoskeletal system defects being the most common group, hypospadias; a defect of the male genital organ was the most prevalent among the sub-group of these defects.

## Introduction

Worldwide, an estimated 134 million births are reported to occur each year of which 7.9 million (6%) are born with at least a major birth defect, mostly affecting the musculoskeletal system and the central system (neural tube defects) [1-3]. Birth defects are defined as abnormalities of intrauterine origin that affect the development of body structures or functions and are present from birth [1-3]. Approximately 30% of these defects are clinically obvious and can be reliably diagnosed either at birth or soon after in the absence of advanced medical techniques [2,4-6]. Birth defects may be classified according to health-related impacts and described as major or minor defects [2]. Alternatively, they may be classified based on anatomical locations and referred to as external or internal defects [2]. Thus, major external structural birth defects (MESBDs) are physical abnormalities detectable at birth that have significant health and developmental impacts on the affected children [1,2]. These defects continue to occur and exert an enormous financial burden to the affected individuals, health services and societal welfare, however, they have been neglected, underestimated and unappreciated as public health problems [1,5,7-9]. Globally, birth defects are among the leading causes of disability-adjusted life years (DALYs), accounting for 25 million DALYs and 2.9% of years of life lived with disabilities [1,2,10,11]. Although birth defects are wide-spread across the world, the highest burden occurs in middle-and low-income countries [1,2,10].

Globally, the prevalence of major external birth defects has been noted to vary by types, severity and geographical regions attributed to data paucity and case under-reporting [5,7,11-14]. In middle-income countries, the prevalence of these defects was estimated at 5.6%, whereas, in low-income countries it was estimated at 6.4%, largely affecting musculoskeletal and central nervous systems [1,5,7,9]. Similarly, defects of the musculoskeletal and central nervous systems were observed to occur more frequently than other MESBDs in Kenya, accounting for 33.9% and 28.6%, respectively [15,16]. In 2010, the prevalence of MESBDs was estimated at 6.3 per 1000 live-births with congenital talipes equinovarus (CTEV), a musculoskeletal disorder being the most common (2.9 per 1000 live-births) in Kenya [10]. Neural tube defects (NTD) followed closely at 0.9 per 1000 live-births for hydrocephalus, 0.5 per 1000 live-births for spina bifida and 0.4 per 1000 live-births for encephalocele [10]. Additionally, hypospadias, a defect of the male genital organ was estimated at 0.9 per 1000 live-births, whereas, cleft lip and imperforate anus were estimated at 0.4 and 0.2 per 1000 live-births, respectively [10]. Notably, spina bifida was reported to have the highest burden of the disease in Kenya, despite a relatively low prevalence estimate [10].

Intrauterine fetal development (embryogenesis) occurs in the first 8 weeks of gestation (first trimester of gestation); a period of great public health importance because of its vulnerability to teratogenicity and the effectiveness of preventive strategies [5,17]. Similarly, fourteen weeks to conception are significant due to the susceptibility of women of reproductive age to teratogens and the appropriateness of effective public health preventive interventions [5,17]. Thus, twelve weeks before conception and eight weeks after conception are critical for effective prevention and control of major external structural birth defects [1,5,18,19]. However, approximately half of pregnancies are usually unplanned, coupled with difficulties in identifying these women during this period and the inability to recognize many pregnancies until the end of the first trimester when the defects have already formed [1,5,18,19]. Despite these observations, little investments have been directed to public health researches, prevention and control activities for these defects in Kenya. Therefore, the objective of this study was to estimate the prevalence of major external structural birth defects in Kiambu County, Kenya.

## Methods

**Study settings and design:** this study was conducted in 13 public hospitals within Kiambu in Kenya. The county´s health department comprised of community health services, 70 dispensaries, 24 health centers, 10 sub-county hospitals and 3 county referral hospitals. The 13 study hospitals consisted of the ten sub-county hospitals; Kihara, Karuri, Wangige, Nyathuna, Lari-Rukuma, Ruiru, Tigoni, Lussigetti, Kigumo and Igegania and the three county referral hospitals; Kiambu, Thika and Gatundu, purposively selected. These hospitals offer reproductive, medical, pediatrics and surgical health services in the 13 sub-counties of Kiambu County; one of the 47 counties in Kenya. Community health services, dispensaries and health centers collaboratively link children born with major external structural birth defects to these hospitals for health care services. Kiambu County is largely urbanized and is the wealthiest among the 47 counties in Kenya. It borders Nairobi County to the south, Muranga and Nyandarua counties to the north, Nakuru and Kajiado counties to the west. It is the second-most densely inhabited county with a population estimate of 2.4 million after Nairobi County (4.3 million) out of 47.5 million nationally [20]. Nakuru County is the third-most heavily populated with an estimate of 2.1 million [20]. All most all births (96.5%) take place in health facilities, whereas about 96.9% of the births are notified for registration for the subsequent issuance of birth certificates in the county [20]. Additionally, about 2.2% of its inhabitants aged five years and above are living with lifelong disabilities [20]. Agriculture (coffee, tea and dairy farming) is the economic mainstay of the county as well as being one of the leading innovative commercial hubs locally. The study adopted a descriptive cross-sectional design to estimate the prevalence of major external structural birth defects; being the best choice of study design for measuring population attributes, providing snapshots of salient public health problems and allowing for results generalization in similar settings.

**Study population and participation eligibility:** the study population (prevalence denominator) comprised all children born to resident women of Kiambu County between January 1^st^, 2014 and December 31^st^, 2018. Cases (prevalence numerator) were defined as live-births with at least one clinically-obvious major external structural birth defect referenced and/or described by a primary healthcare provider, either in the delivery rooms or neonatal units. These defects were considered for this study because they were easily recognizable visually or through physical examination at birth or shortly after birth by a healthcare provider. Additionally, case ascertainment was less likely to be affected by regional differences in referral and medical treatment compared to other anomalies. Primary health care providers consisted of midwives, medical officers and obstetricians in delivery-rooms, whereas, in neonatal units, the primary providers comprised nurses, midwives, medical officers, clinicians and pediatricians. The choice of the study population helped to provide a glimpse of the public health magnitude of these defects in Kiambu County. The results acted as a pointer to the “silent epidemic” and were intended to inform public health planning, policy decisions and actions on public health surveillance and birth defect-specific interventions.

**Exclusion criteria:** the study excluded stillbirths, new-born to non-resident women of Kiambu County and new-born with a clinically undetectable or unobvious birth defect at birth or soon after birth. Additionally, externally occurring structural birth defects with no significant medical and financial implications were excluded from the study.

**Sources and collation of numerator data:** the study extracted numerator data from maternity files, maternity registers, neonatal inpatient files and neonatal daily-bed returns. Maternity files contain records about women admitted in delivery rooms for childbirth. Information captured in these files is demographic, social, medical, surgical and reproductive history, admission findings, intra-partum and postpartum care. These files have summary sections for recording birth defects visually identified at birth or soon after birth by the primary health care providers. Summaries of the maternity files are then entered in maternity registers for the compilation of hospital reports and used as complementary sources of data. Similarly, neonatal inpatient files contain maternal information described in the maternity files above and information of the neonates. Information about neonates is further summarized in daily-bed returns and used as supplementary sources of data. Before data collection, six research assistants (RAs) were recruited and trained in data extraction techniques to ensure that the abstraction process was carried out in a standardized manner. To obtain the numerator data, medical-related records described above were reviewed over the five-year study period. All MESBDs referenced and/or described by primary health care providers were extracted and entered in a predefined data abstraction tool. Information captured during data extraction included names of the study hospitals, sources of data, sub-county of residence, dates of admission, dates of birth, sex of the newborn children, definitions or descriptions of the birth defects, referrals from homes, peripheral health facilities and to other health facilities.

On the other hand, annual prevalence denominators (the number of registered live-births) was drawn from the Kenya Vital Statistics Report, 2017 (KVSR 2017); a publication of Civil Registration Services [21]. The registration process begins with the notification of births by health workers and assistant chiefs as civil registration assistants who enter the information of the new-born children in birth notification registers. The birth notification register is filled in duplicate known as acknowledgement of birth notification; the counterfoil copies are retained by registration assistants, whereas, the original slips are given to the parents or next of kin for the subsequent issuance of official birth certificates. These registers contain variables such as sex (male/female), type of birth (single/multiple) and nature of birth (alive/dead).

**Ethical considerations:** ethical approval was obtained from Kenyatta National Hospital (KNH)-University of Nairobi (UoN) Ethics Review Committee (Ref. No: KNH-ERC/A/44). Data collected were also de-identified using anonymous codes.

**Statistical analysis:** data obtained from secondary data abstraction tools were double-entered into an excel-spreadsheet by two independent data clerks to minimize errors. The principal investigator exported the validated dataset to Stata software, version 14 (Stata Corporation, College Station, Texas, USA) for cleaning and analyses. The prevalence estimates of major external structural birth defects were calculated as the number of cases (numerator) divided by the total number of live-births (denominator) using the formula below:

$$Prevalence=\frac{Numerator}{Denominator}x100,000\;live-births$$

Associated 95% binomial exact confidence intervals were also computed and expressed per 100,000 live-births. Summary results were presented in proportions using frequency tables and graphs to show the distribution of these defects in the county.

## Results

The study observed 362 cases categorized into six groups and 29 sub-groups of major external structural birth defects (Table 1). Defects of the musculoskeletal system (57.46%) were the most frequent of the six groups of MESBDs followed by defects of the central nervous system (17.13%), orofacial defects (13.26%) and defects of the genital organs (11.05%) (Table 1). Additionally, anal and ocular defects were observed in the county during the study period. Congenital talipes equinovarus (73.08%), limb reduction defects (9.61%), gastroschisis (5.77%) and omphalocele (3.85%) were among the musculoskeletal system defects observed during the study period (Table 1). Notably, limb reduction defects consisted of unspecified lower limb reduction (5.77%), unspecified upper limb reduction defects (1.92%), phocomelia (0.96%), amelia (0.48%) and congenital femoral deficiency (0.48%) (Table 1). Of the musculoskeletal system defects, abdominal muscle defects (gastroschisis and omphalocele) accounted for 9.62%, whereas, congenital knee defects consisting of arthro-onchyo-dysplasia (0.96%), congenital patella aplasia (0.48%) and congenital patella dysplasia (1.92%) accounted for 3.36% (Table 1).

**Table 1:** proportions of groups and sub-groups of MESBDs in Kiambu County, 2014-2018

Groups of MESBDs	Sub-groups of MESBDs	Frequency	Percent
**Musculoskeletal system defects**		**208**	**57.46**
	Congenital talipes equinovarus	152	73.08
	Reduction defects of the limbs	20	9.61
	Clubbed hand	7	3.37
	Ectrodactyly	1	0.48
	Congenital knee defects	7	3.37
	Conjoint twins	1	0.48
	Gastroschisis	12	5.77
	Omphalocele	8	3.85
**Central nervous system defects**		**62**	**17.13**
	Anencephaly	19	30.65
	Hydrocephaly	16	25.81
	Spina bifida	10	16.13
	Microcephaly	4	6.45
	Craniorachischisis	2	3.23
	Encephalocele	2	3.23
	Meningocele	2	3.23
	Neurological defect	1	1.61
	Sacrococcygeal teratoma	1	1.61
	Craniosynostosis	1	1.61
	Congenital scoliosis	4	6.45
**Oral-facial clefts**		**48**	**13.26**
	Cleft lip with palate	37	77.08
	Cleft lip without palate	7	14.58
	Cleft palate	4	8.33
**Defects of genital organs**		**40**	**11.05**
	Hypospadias	33	82.50
	Epispadias	4	10.00
	Unformed genitalia	1	2.50
	Malformed penis	2	5.00
**Defects of eye**		**2**	**0.55**
	Anophthalmia	1	50.0
	Congenital cataract	1	50.0
**Defects of anus**		**2**	**0.55**
	Imperforate anus	2	100.00
**Total**		**362**	**100.00**

**Figure 1:**
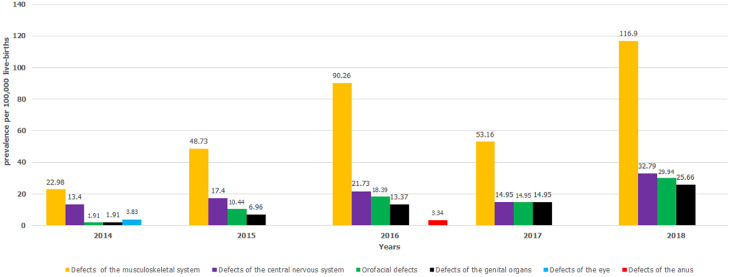
bar chart for the prevalence of six groups of MESBDs in Kiambu County, 2014-2018

**Figure 2:**
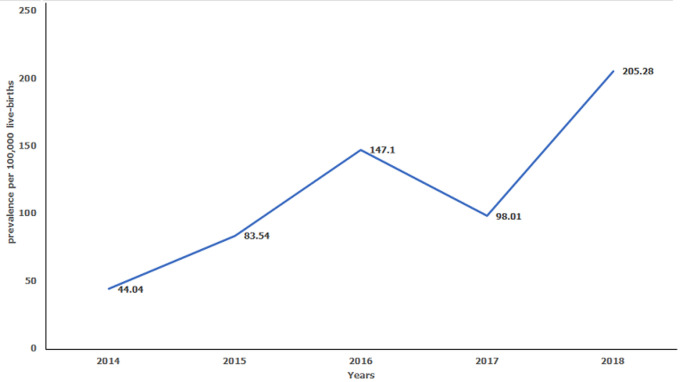
line graph for the prevalence of six groups of MESBDs in Kiambu County, 2014-2018

The study further showed anencephaly (30.65%), hydrocephalus (25.81%) and spina bifida (16.13%) were common among the defects of the central nervous system (Table 1). Conspicuously, hypospadias, a defect of the male genital organ accounted for 82.5% of all defects of genital organs (Table 1). The prevalence estimates for the defects of musculoskeletal and central nervous systems ranged from 22.98 (95% CI: 11.87-40.13) to 116.90 (95% CI: 92.98-145.08) per 100,000 live-births and 13.40 (95% CI: 5.39-27.61) to 32.79 (95% CI: 20.79-49.19) per 100,000 live-births during the study period, respectively (Table 2). Similarly, the study showed a remarkable variation in annual prevalence estimates of the six groups of MESBDs (Figure 1). There was a steady annual increase in the prevalence estimates of the six groups of MESBDs ranging between 44.04 (95% CI: 27.92-66.07) in 2014 and 205.28 (95% CI: 173.15-241.64) per 100,000 live-births in 2018, despite a slight decline in 2017 estimated at 98.01 (95% CI: 74.62-126.41) per 100,000 live-births (Figure 2).

**Table 2:** prevalence of MESBDs per 100,000 live-births in Kiambu County, 2014-2018

Year	Live-births (N)	Groups of major external structural birth defects	Cases (n)	Prevalence per 100,000 live-births	95% confidence interval
2014	52229	Defects of the musculoskeletal system	12	22.98	11.87-40.13
		Defects of the central nervous system	7	13.40	5.39-27.61
		Orofacial defects	1	1.91	0.0485-10.67
		Defects of the genital organs	1	1.91	0.0485-10.67
		Defects of the eye	2	3.83	0.46-13.83
		Defects of the anus	-	-	-
2015	57456	Defects of the musculoskeletal system	28	48.73	32.39-70.43
		Defects of the central nervous system	10	17.40	8.35-32.01
		Orofacial defects	6	10.44	3.83-22.73
		Defects of the genital organs	4	6.96	1.90-17.82
		Defects of the eye	-	-	-
		Defects of the anus	-	-	-
2016	59824	Defects of the musculoskeletal system	54	90.26	67.82-117.76
		Defects of the central nervous system	13	21.73	11.57-37.16
		Orofacial defects	11	18.39	9.18-32.90
		Defects of the genital organs	8	13.37	5.77-26.35
		Defects of the eye	-	-	-
		Defects of the anus	2	3.34	0.41-12.08
2017	60198	Defects of the musculoskeletal system	32	53.16	36.36-75.03
		Defects of the central nervous system	9	14.95	6.84-28.38
		Orofacial defects	9	14.95	6.84-28.38
		Defects of the genital organs	9	14.95	6.84-28.38
		Defects of the eye	-	-	-
		Defects of the anus	-	-	-
2018	70147	Defects of the musculoskeletal system	82	116.90	92.98-145.08
		Defects of the central nervous system	23	32.79	20.79-49.19
		Orofacial defects	21	29.94	18.53-45.76
		Defects of the genital	18	25.66	15.21-40.55
		Defects of the eye	-	-	-
		Defects of the anus	-	-	-

## Discussion

To our knowledge, this is the first cross-sectional study conducted to estimate the prevalence of major external structural birth defects in Kiambu County. Despite the rarity of these defects, their effects are adverse, thus underscore the assembly and analysis of locally available data to understand the public health magnitude of the problem [22]. This could stimulate public health researches, inform birth defect-specific surveillance programs and interventions aimed at controlling and preventing the occurrence of these defects [22]. Our study observed six groups of MESBDs affecting the musculoskeletal system, central nervous system, orofacial organs, genital organs, ocular and anal organs, thus contributing to the worldwide empirical debate on MESBDs as a salient global problem awaiting explosion.

Defects of the musculoskeletal system and those of the central nervous system were shown by the study as the most prevalent in Kiambu County. These findings are indeed consistent with the results of other studies carried out in the region, such as Ethiopia [2,6,15,16,23]. The proportional contribution of musculoskeletal system defects to the overall prevalence of MESBDS was however exceedingly high; our study observed almost two-thirds (57.46%), whereas, as a study conducted in 1984 reported one-third (33.9%) of the MESBDs in Kenya [15]. These findings point to possible increased exposure of women of reproductive age to teratogenic chemicals, metals and preconception obesity that have been associated with the defects of the musculoskeletal system [24,25]. Because Kiambu is largely an agricultural county, there is an increased likelihood of exposure to pesticide-related chemicals and metals among women of reproductive age. Some studies, however, reported defects of the central nervous system as the most common among MESBDs, pointing to the ineffectiveness of public health prevention and control strategies for such defects in other regions [7,9]. In Kenya, studies have shown a steady decrease in the prevalence of neural tube defects accounting for 28.6% and 16.15% of all MESBDs in 1984 and 2015, respectively [15]. Similarly, a relatively low (17.13%) contribution of neural tube defects to MESBDs was observed in our study, further demonstrating a decline in the trend of NTDs locally. Increased pre-conception and post-conception folic-serum levels are known to effectively prevent the occurrence of neural tube defects. Therefore, the decline in the prevalence of neural tube defects being observed could have been as a result of increased iron-folic acid supplementation by pregnant women in Kenya [1,2,6,7,17,26]. The trends of neural tube defects and other major external structural birth defects could be reversed further if effective public health interventions are strictly implemented at least twelve weeks pre-conception and eight weeks post-conception.

Even though this study reported 29 sub-groups of the six groups of MESBDs with musculoskeletal and central nervous defects as the most common among the groups in general, hypospadias (82.50%); a male genital organ defect, was the most frequently occurring sub-group of MESBDs. Although many studies have not been reporting findings on defects of genital organs, this study further showed epispadias; another defect of the male genital organ was similarly common in the county. This could be suggestive of a constant prevalence of the most common risk factors for hypospadias and other genital defects such as advanced maternal age (35 years) and BMI (>26) [27]. Notably, cleft lip with the palate (77.08%) was the second most common sub-group of MESBDs in the county. This also mimicked findings of other studies showing cleft lip with the palate and cleft lip without palate as the most prevalent defects among orofacial clefts [27,28]. Instead, congenital talipes equinovarus (57.46%); one of the defects of the musculoskeletal system was unexpectedly reported third among the most prevalent sub-groups of MESBDs. Nonetheless, this observation corroborated other studies reporting congenital talipes equinovarus as being the most common among defects of the musculoskeletal system [1,2,15]. Remarkably, limb reduction defects were observed in this study as the second most common among musculoskeletal system defects, however, the locally existing empirical literature has not been reporting proportions of such defects. Anencephaly (30.65%) and hydrocephalus (25.81%); defects of the central nervous system were fourth and fifth, respectively. Although other study findings reported hydrocephalus as the most commonly occurring NTD followed by spina bifida and encephalocele; anencephaly emerged as the most prevalent among NTDs in our study [10]. Similarly, this study noted a decrease in cases of encephalocele (3.23%) [10]. Anencephaly is a highly fatal neural tube defect known to significantly contribute to prenatal deaths and lead to few live-births with anencephaly, however, this was not the case in this study [1,2,16,22,29]. Instead, many cases of anencephaly were observed pointing to possible increased prevalence of risk factors specific to anencephaly and not NTDs in its entirety. This phenomenon underpins endeavors to further researches to determine other risk factors for anencephaly and the importance of routine autopsies on all stillbirths to establish the causes of such deaths for purposes of improving prevalence estimations of MESBDs.

Certain limitations were however noted during the study period; first and foremost, there were inadequate stores and shelves for the medical records leading to heaped pools of records not necessarily arranged in an ordered manner and sometimes leading to defaced records. Secondly, medical records used in this study were not designed for epidemiological studies, therefore, researchers perused over many pages of maternity files to identify summary sections of the files where major external structural birth defects were defined, referenced, or described. Additionally, although the files were accessible in one of county referral hospitals, summary sections of maternity files for recording MESBDs were missing, therefore no records were abstracted for the five-year study period in this facility. In 2017, maternity files in another county referral hospital were not accessed because they were relocated to an unknown place within the hospital, explaining a decline in prevalence estimates observed (Figure 2). Further, cases described as congenital anomalies, gross congenital anomalies and multiple congenital anomalies were excluded as part of the study numerator because we were unable to distinguish them for categorization into the six groups and sub-groups of the major external structural birth defects. Additionally, stillbirths were ineligible for inclusion in the study because their causes were unknown to the researchers. The factors described above certainly contributed to underestimations and variations of the prevalence estimates of MESBDs in the county.

## Conclusion

This was the first study to estimate a region-specific prevalence of major structural birth defects in Kenya. Despite the limitations of the study and the fact that defects of the musculoskeletal, central nervous system were the most frequent groups of major external structural birth defects, hypospadias; a defect of the male genital organ was the most prevalent among the 29 sub-groups of the defects in the county. Additionally, anencephaly, was the most commonly occurring neural tube defect despite being associated mostly with stillbirths. This observation pointed at the possibility of constant exposure to women of reproductive age to potential risk factors for a myriad of major external structural birth defects. In the absence of effective prevention and control measures, accessible corrective and rehabilitative services for major external structural birth defects; adverse health effects, psychosocial impacts, developmental challenges and reduced economic productivity arising from lifelong disabilities are inevitable. Establishing county-specific and national surveillance systems for major external structural birth defects will be of great public health importance in understanding the public health magnitude of these defects, regionally and nationally. Lastly, we recommend that future studies should investigate risk factors for major external structural birth defects in Kiambu County with a view of cascading similar studies across the country for purposes of informing designs and formulations of defect-specific surveillance, prevention and control strategies.

### What is known about this topic

- There is a wide-spread limitation of local data for major birth defects leading to underestimation of their prevalence;
- The prevalence of major external structural birth defects is known to vary from region to region and from one period to the other;
- The burden associated with major external structural birth defects includes childhood morbidity, childhood mortality, lifelong disabilities, reduced quality of life, reduced life expectancy and reduced economic productivity.

### What this study adds

- Overall, the study showed defects of the musculoskeletal and central nervous systems were the most frequent among the six groups of MESBDs in the county. However, hypospadias (82.50%), cleft lip with the palate (77.08%) and congenital talipes equinovarus (73.08%) were the most commonly occurring among the 29 sub-groups of MESBDs in the county;
- Although anencephaly is the leading cause of stillbirths associated with severe birth defects, it was among the most prevalent (30.65%) among the sub-groups of MESBDs among live-births and the highest among defects of the central nervous system;
- The study showed a spiraling trajectory of major external structural birth defects during the five-year study period.
